# Measuring and analyzing tissue specificity of human genes and protein complexes

**DOI:** 10.1186/1687-4153-2011-5

**Published:** 2011-08-04

**Authors:** Dorothea Emig, Tim Kacprowski, Mario Albrecht

**Affiliations:** 1 Max Planck Institute for Informatics, Campus E1.4, 66123 Saarbrücken, Germany

**Keywords:** gene expression, protein interaction, tissue specificity

## Abstract

Proteins and their interactions are essential for the survival of each human cell. Knowledge of their tissue occurrence is important for understanding biological processes. Therefore, we analyzed microarray and high-throughput RNA-sequencing data to identify tissue-specific and universally expressed genes. Gene expression data were used to investigate the presence of proteins, protein interactions and protein complexes in different tissues. Our comparison shows that the detection of tissue-specific genes and proteins strongly depends on the applied measurement technique. We found that microarrays are less sensitive for low expressed genes than high-throughput sequencing. Functional analyses based on microarray data are thus biased towards high expressed genes. This also means that previous biological findings based on microarrays might have to be re-examined using high-throughput sequencing results.

## Introduction

It is essential for human systems biology and medicine to understand the tissue specificity of expressed genes and their products, which are involved in important cellular processes and diseases. Over the last years, many studies were based on the freely available Novartis Gene Atlas data to investigate the tissue specificity of human gene expression and its biological impact on protein expression and protein interaction networks [1,2]. The Gene Atlas data consists of comprehensive gene expression datasets for a wide variety of tissues and cell lines [3]. However, these data were already published in 2004, and the microarrays employed to obtain the data were of low probe density and specifically designed to measure genes that were assumed to exist at that time. This raises the question whether these relatively old datasets should still be regarded as reliable source for tissue specificity of human genes. A more recently developed microarray is the Affymetrix Exon Tiling Array, which has been developed to measure exon expression rather than gene expression [4]. Its probe density per gene is much larger than the microarray technology used to generate the Gene Atlas. Furthermore, the advent of next-generation sequencing machines allows further technological advances in accurate transcriptome measurements [5].

In the following, we explore three tissue-dependant gene expression datasets produced by microarray technologies and high-throughput sequencing of RNA. We first study the detection sensitivities of the technologies and compare the measured gene expression datasets. Furthermore, we investigate protein interactions to identify tissue-specific and housekeeping interactions. Last, we utilize expression data for the detection and comparison of tissue-specific protein complexes and analyze to what extent functional implications on tissue specificity depend on the applied expression detection method.

## Materials and methods

### Databases and identifier unification

All analyses are based on the Ensembl database, version 52 (genome build hg18) [6]. Gene and protein identifiers of all data sources were unified by mapping them to Ensembl gene identifiers via Ensembl BioMart [7].

### Tissue samples

We downloaded the raw Novartis Gene Atlas data from GEO (GSE1133) together with probeset-to-gene annotations for the GNF1H and Affymetrix U133A arrays. The data contains samples for 79 human tissues and cell lines. For the Affymetrix Exon Array, we downloaded sample data for 11 tissues as provided by Affymetrix, with three assay replicates for each tissue. RNA-sequencing data for 15 tissues and cell lines was obtained from the supplementary data provided by Wang *et al. *[8]. Five human tissue samples were contained in all three expression datasets: heart, liver, testis, skeletal muscle, and cerebellum.

### Probeset to gene mapping

Probesets for the Gene Atlas arrays were mapped to Ensembl genes using all gene identifiers as given in the GEO probeset-to-gene annotation files. For the GNF1H array, we were able to map 8,875 probesets to 6,086 Ensembl genes, out of which 5,943 encode proteins. For the Affymetrix U133A array, we were able to map 21,778 probesets to 12,489 Ensembl genes, out of which 12,448 encode proteins. The Gene Atlas data are based on both microarrays and consists of a total of 16,989 distinct protein-coding genes.

The Exon Array probesets were mapped to Ensembl genes according to the genomic coordinates of the probesets as given in the NetAffx release 28 [9]. Altogether, the probesets could be mapped to 20,444 protein-coding genes.

### Gene expression estimates

The raw Novartis Gene Atlas data were normalized using the Affymetrix Expression Console software. All samples were normalized together by applying the MAS5.0 algorithm with default parameters. The resulting presence/absence calls (P-/A-calls, automatically derived by MAS5.0 from computed detection *p*-values) for the probesets were then used to identify genes expressed in the respective samples. For simplification, we treated marginal calls (M-calls) as present. We regarded a probeset as being present in a sample if it was present in at least one of the two replicates. If more than one probeset mapped to one gene, we required at least one of the probesets to be present for gene expression.

The Exon Array data were processed using AltAnalyze with default parameters [10]. AltAnalyze computes a detection *p*-value for every Ensembl gene in each of the three replicates per sample. The *p*-values are derived using the DABG ('detection above background') method, which is the standard method for computing P-/A-calls for Exon Arrays. We obtained gene presence and absence calls by taking the median of the three *p*-values for every gene in each sample, and set the presence *p*-value threshold to 0.05, which is the recommended threshold for DABG *p*-values.

Gene expression estimates (RPKM values) for the RNA-sequencing data were obtained from Wang *et al. *[8]. We chose a very conservative expression threshold and treated all genes having an RPKM value ≥ 1 as present and all others as absent [5]. In contrast to the other tissues with a single sample each, six different samples were available for cerebellum. To obtain a single RPKM value per gene in cerebellum, we took the mean of these expression estimates and regarded genes as expressed if their mean RPKM values were ≥ 1.

### Comparison of detection calls

Although the three datasets contain many tissue and cell line samples, the overlap consists of five tissues only. Thus, we defined a gene to be tissue-specific if it is expressed in exactly one of these five tissues.

The gene presence and absence calls amount to a binary classification of gene expression results that does not take expression levels into account. Therefore, we used the Matthews correlation coefficient (MCC) to compute pairwise correlations between the datasets. The MCC is computed as follows:

Here, TP is the number of true positives, i.e. genes classified as expressed in both datasets. TN is the number of true negatives, i.e. genes classified as not expressed in both datasets. FP is the number of false positives, i.e. genes classified as expressed only in the one, but not the other dataset. FN is the number of false negatives, i.e. genes classified as expressed only in the other dataset.

### Protein interaction data

We obtained a human protein interaction network from a recent study by Bossi and Lehner [1]. The protein interactions had been compiled from more than 20 data sources and required to have experimental evidence of physical interaction. We mapped all proteins to Ensembl gene identifiers. We kept a protein interaction if both interacting partners could be mapped and had expression estimates in all datasets. This gave 60,760 interactions.

### Protein complex data

Human protein complexes were obtained from PDB and CORUM (downloaded July 2009) [11,12]. We mapped all complex members to Ensembl gene identifiers. We kept only those complexes for which all proteins could be mapped and had gene expression estimates in all three expression datasets. We also required the complexes to be composed of at least three different proteins and removed duplicates contained in CORUM and PDB data. This resulted in 572 distinct protein complexes.

## Results and discussion

### Gene expression analysis

We first extracted those protein-coding genes contained in all three expression datasets, a total of 14,718 Ensembl genes, to compare their presence/absence calls (i.e. expression detected or not). We find that RNA-sequencing and Exon Array data have a comparatively high agreement in their presence and absence calls, while the Novartis Gene Atlas shows inverse calls for many genes. More precisely, the correlation between the RNA-sequencing and Exon Array data is clearly higher than the correlation of any of these datasets to the Gene Atlas data (MCC was used for all analyses). On average, the correlation between RNA-sequencing and Exon Array data is 0.56, with a maximum of 0.61 in liver and a minimum of 0.44 in testis. The average correlation between the Gene Atlas and RNA-sequencing data is 0.27 and between Gene Atlas and Exon Array data 0.28. The respective maximal correlations are 0.31 (in liver) and 0.32 (in testis), and the minimal correlations 0.18 and 0.20 (both in muscle).

**Figure 1:**
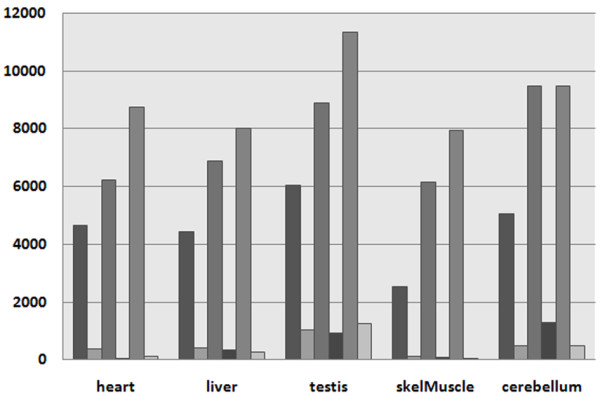
**Number of all expressed genes and the tissue-specific fraction in each tissue as detected by the Gene Atlas (2 left bars), Exon Array (2 middle bars), and RNA-Seq (2 right bars)**.

RNA-sequencing is the most sensitive method for detecting gene expression. Figure 1 shows that, for each tissue (except for cerebellum), the number of expressed genes is the highest when using RNA-sequencing, a finding that is in agreement with a recent study by Ramskold *et al. *[5]. Of course, the number of expressed genes depends on the RPKM expression threshold to some extent. However, the study by Ramskold and colleagues showed that an RPKM threshold below our choice still yields reasonable results. Thus, lowering the threshold would increase the number of expressed genes using RNA-sequencing even further.

As seen from the correlation of the A-/P-calls above, the Gene Atlas arrays are not able to detect many of the genes found expressed according to the Exon Array and RNA-sequencing. The number of tissue-specific genes (expressed in exactly one of the five tissues) is low for all methods. The fewest tissue-specific genes are detected in skeletal muscle and the highest in testis. We also compared the actual genes found to be expressed according to the different methods. We observed a high agreement of genes with P-calls for RNA-sequencing and Exon Arrays, with the lowest agreement (37%) in skeletal muscle and the highest (56%) in cerebellum. The Gene Atlas, however, is not able to detect many of these genes and, on average, shows a low agreement with the other datasets.

A closer look at the tissue specificity of expressed genes reveals that the gene expression detection results vary significantly between the datasets and across tissues. While RNA-sequencing detects more than 6,000 genes (41% of all shared genes) to be expressed in all tissues, the Exon Array identifies only about 4,500 genes (31%) and the Gene Atlas indicates only about 1,500 genes (10%) to be expressed in all tissues. The reverse can be observed for those genes not expressed in any of the five tissues: RNA-sequencing identifies the lowest number of absent genes (approx. 2,100), while the Gene Atlas is not able to detect more than 6,000 genes. For genes expressed in one to four tissues, the numbers are very similar for all datasets.

These results demonstrate clearly that more genes are widely expressed than previously thought and that tissue expression studies will need to be re-examined using the novel RNA-sequencing method [5]. Obviously, microarrays are less sensitive regarding gene expression detection than RNA-sequencing methods. Statistical methods used for normalizing microarray data often cannot distinguish between very low gene expression and experimental noise. Therefore, it is likely that low expression is mistakenly reported as noise and thus the respective gene is regarded as not expressed. RNA-sequencing methods, which are based on read-to-gene mappings, can reliably detect genes at very low expression levels.

We also compared the detection sensitivity of RNA-sequencing and the microarrays. For each tissue, we first extracted all shared genes with an RPKM ≥ 1 from the RNA-sequencing data. We found that RNA-sequencing detects a high number of genes expressed at low levels. Next, we investigated the fraction of these genes that are also detected as expressed by the microarray methods and annotated the respective RPKM values to them. We observed that, for all tissue samples, the Exon Array identifies a greater number of genes expressed at low levels (with a low RPKM value according to the RNA-sequencing data) than the Gene Atlas. This suggests that the high probe density of the Exon Array can partly compensate the errors due to experimental noise.

### Tissue specificity of protein interactions

**Figure 2:**
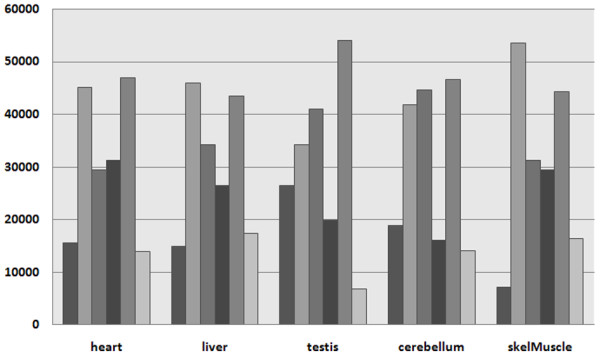
**Histogram of the numbers of present and absent protein interactions in each tissue**. The two leftmost bars show presence and absence according to the Gene Atlas, the third and fourth bars according to the Exon Array, and the two rightmost bars according to RNA-sequencing.

Gene expression often leads to the production of proteins in the cells. Therefore, we re-examined a study regarding the tissue specificity of physical protein interactions, which was based on the Gene Atlas [1]. In this study, a high number of tissue-specific protein interactions was reported, which mainly occurred due to the interaction of a tissue-specific protein with a housekeeping protein. Using the Gene Atlas data, we can reproduce these findings. However, Figure 2 shows that the number of protein interactions occurring in the tissues rapidly grows when applying the Exon Array or RNA-sequencing data. While the number of absent protein interactions compared to present ones is always higher when applying the Gene Atlas, the results are reversed using the other methods. This finding suggests that fewer protein interactions are tissue-specific than assumed previously, and relatively few protein interactions contribute to tissue-specific functions.

### Tissue specificity of protein complexes

**Figure 3:**
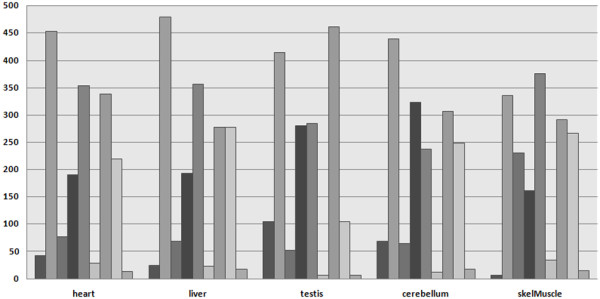
**Expression of protein complexes in each tissue according to the Gene Atlas (three leftmost bars), Exon Array (three middle bars), and RNA-Seq (three rightmost bars)**. The respective three bars are ordered according to complete expression, partial expression, and complete absence of the protein complexes.

Additionally, we investigated whether microarrays and RNA-sequencing are able to detect the expression of protein complexes in different tissues. We distinguished between completely expressed complexes (all involved genes are expressed), partially expressed complexes (at least one of the involved genes is not expressed, but we require the partial complex to consist of at least two expressed proteins), and completely absent complexes (at most one involved genes is expressed). As shown in Figure 3, RNA-sequencing is the most sensitive method, and the highest number of completely expressed protein complexes is found in all tissues. In contrast, the Exon Array identifies fewer complexes, and the Gene Atlas hardly detects any complexes as completely expressed. Since the detection sensitivity of the Gene Atlas has been shown to be the lowest, we expected to find few completely expressed complexes. However, the detection rate for protein complexes is even lower than thought, with only 0.01% in skeletal muscle (compared to 51% using RNA-sequencing). Conversely, it is interesting that the number of completely absent complexes is low for all methods, suggesting that most of them contain high expressed gene products detectable by all methods.

To compare the expression measurements of the microarrays and RNA-sequencing, we computed their correlations regarding the detection of protein complexes. For this purpose, we calculated the detection percentage for each complex and each measurement method. Detection percentages of 0% and 100% indicated that the complex is completely absent and present, respectively, while everything in between was a partially expressed complex. As for the gene expression correlation, the expression correlation for protein complexes is clearly higher for RNA-sequencing and the Exon Array than for any method correlated to the Gene Atlas. The average correlation for RNA-sequencing and the Exon Array is 0.66, with a maximum of 0.73 in muscle and a minimum of 0.48 in testis. For the Gene Atlas and RNA-sequencing as well as the Gene Atlas and the Exon Array, the average correlation is 0.31 in both cases, with a minimum of 0.23 (muscle) and a maximum of 0.39 (cerebellum), and a minimum of 0.26 (cerebellum) and a maximum of 0.36 (testis), respectively.

## Conclusions

Our analysis revealed that gene expression varies depending on the method used for detection. We found that, using RNA-sequencing technologies, a considerably larger number of genes is found to be widely expressed than previously thought and that many of the detected genes are expressed at low levels. Using the very common, yet low-density, 3' microarrays, we were not able to detect many of these genes. However, it is remarkable that the Exon Array results correlate well with the RNA-sequencing results, which suggests that the high probe density of this microarray is partially able to identify low gene expression.

In addition, we integrated the gene expression results obtained by the different technologies with protein interactions and protein complexes to investigate to what extent the discovered differences in gene expression might affect the outcome of functional analyses. We observed that, in case of 3' microarrays, the overall number of protein interactions and complexes expressed in each tissue is low and that many interactions and complexes are classified as highly tissue-specific. In contrast, based on RNA-sequencing, a considerably larger number of protein interactions and complexes is found per tissue, and we classified much fewer of them as tissue-specific. These results indicate that previous functional analyses that relied on 3' microarrays should be reconsidered because they suggested a large number of tissue-specific proteins and interactions. However, these earlier findings were likely biased towards highly expressed genes and thus could not provide accurate insight into tissue specificity and its functional impact.

## List of Abbreviations

DABG: detection above background; MCC: Matthews correlation coefficient.

## Competing interests

The authors declare that they have no competing interests.
